# The Impact of Cognitive Ability and Self-Control on Middle School Students’ Comprehensive Academic Performance—The Moderating Role of Learning Plan

**DOI:** 10.3390/jintelligence13080092

**Published:** 2025-07-24

**Authors:** Yueqi Shi, Junyao Yang

**Affiliations:** Institute of Education, University of Science and Technology Beijing, Beijing 100083, China; m202411130@xs.ustb.edu.cn

**Keywords:** cognitive ability, self-control, learning plan, academic performance, structural equation modeling

## Abstract

In the educational context, understanding factors affecting secondary school students’ academic performance is crucial. This study aimed to explore impacts of cognitive ability, self-control, and study plans and their interactions. Using data from 1477 students aged 15–18, the moderated mediation model was applied. Results verified a positive link between cognitive ability and performance, found self-control as a mediator, and revealed study plans’ moderating effect. In conclusion, these elements play key roles, providing a theoretical basis for educators to optimize strategies and promote students’ overall development.

## 1. Introduction

In the domains of education and psychology, the comprehensive academic performance of middle school students and their personal growth and development trajectories have long been central research themes, garnering substantial attention within scholarly circles. For an extended period, when the academic community investigates the factors influencing academic performance, while traditionally focusing on intellectual-level factors as key elements, there is also growing recognition of the significant roles played by non-cognitive factors—with persistence and self-discipline serving as typical exemplars—in the learning process and even in individuals’ pathways to success. These non-cognitive elements cannot be overlooked in scholarly analyses of academic achievement determinants.

In China, although the educational evaluation system is gradually diversifying, academic performance remains the primary criterion for evaluating students’ academic achievements. When enrolling students, higher education institutions often take academic performance as the main reference basis. Therefore, many Chinese scholars have been constantly exploring various factors that influence high school students’ academic performance, especially paying attention to the interaction between cognitive abilities and non-cognitive abilities. Existing research, however, has predominantly centered on the relationships between intellectual factors and non-intellectual constructs—such as willpower, self-discipline, and responsibility—and their associations with academic outcomes. A notable gap in these studies lies in the lack of explicit definitions and empirical correlations delineating the specific influences of non-intellectual factors. Moreover, both domestic and international scholars have rarely explored the role of planning as a determinant of students’ academic performance. Empirical investigations examining self-control as an intermediary variable in this relationship remain comparatively limited, representing an under-researched domain in the extant literature.

Consequently, this study delved deeply into the specific impacts of cognitive ability, the self-control factor among non-cognitive abilities, and learning plan and other factors on academic performance, and also explained the potential mechanisms of the interaction among these factors.

## 2. Literature Review and Research Hypotheses

### 2.1. The Influence of Cognitive Abilities

Cognitive ability pertains to the diverse functions and processes through which the human brain performs information processing. It encompasses a range of specific capabilities, including perception, memory, thinking, reasoning, and decision-making, as defined by Goldstein (2018). Intelligence, in contrast to cognitive ability, constitutes a more comprehensive concept. Commonly, it is characterized as an individual’s aptitude to comprehend intricate concepts, adapt to the environment, draw lessons from experiences, and solve problems, as defined by Gardner (2011). Cognitive ability focuses on particular mental functions and processes, such as attention, memory, and thinking patterns, and serves as a foundational component. Conversely, intelligence represents a more comprehensive construct, embodying an individual’s overall cognitive competence and the capacity to adapt to the environment, as stated by Gerrig et al. (2010). In the present study, cognitive ability is conceptualized as an integral part of intelligence and is further operationalized into five dimensions: working memory ability (MA), representation ability (RA), information processing ability (IPA), logical reasoning ability (LRA), and thinking conversion ability (TCA). Notably, these five aspects are closely correlated with the academic performance of high school students.

The dimension division of cognitive ability in this study (MA, IPA, RA, LRA, TCA) is highly consistent with the theoretical framework of CHC (McGrew 2009) and is empirically supported by local studies in China (Chen and Zhao 2016). The specific mapping relationship is as follows: working memory (MA) corresponds to the working memory capacity (Gwm) of CHC; logical reasoning (LRA) and thought transformation (TCA) jointly reflect fluid reasoning (Gf); information processing (IPA) directly matches processing speed (Gs); and representational ability (RA) is associated with visual processing (Gv). The low correlation between factors (such as r = 0.025 between RA and MA) conforms to the characteristics of the CHC hierarchical model: each dimension retains an independent variance, and this has been verified in exploratory factor analysis. The low correlation precisely indicates that this scale effectively distinguishes the independent cognitive dimensions of CHC.

In recent years, scholarly research on the correlation between cognitive ability and students’ academic performance has primarily focused on examining the ways in which cognitive ability impacts academic attainment and the various underlying mechanisms. Cognitive ability directly influences academic performance. For example, scholars Sathishkumar and Sengamalam (2024) explored the impact of logical reasoning ability on high school students’ mathematics performance and found that students with stronger logical reasoning ability tend to achieve better results in this subject. Working memory ability is one of the important factors explaining individual differences in academic performance. Students with high working memory ability tend to perform better in liberal arts subjects, such as in English listening comprehension (Cao 2023), and in different types of reading comprehension questions (He and Liu 2024). The cultivation of representation ability helps students to understand abstract problems and objects in mathematics more deeply. Based on this research finding, China’s Compulsory Education Curriculum Standards for compulsory education have also included the cultivation of students’ mathematical representation ability in teachers’ teaching requirements (Zheng 2024). Information processing ability mainly refers to students’ understanding of and processing speed for acquired text content. This ability is positively correlated with the successful solution of practical text problems among middle school students (He 2022). Thinking conversion ability is manifested in an individual’s ability to quickly adjust their cognitive strategies and problem-solving strategies when facing different situations, to use new methods to solve problems, and to adapt to new environments. This ability enables students to flexibly adapt to different learning environments and requirements, effectively manage learning time, integrate interdisciplinary knowledge, and maintain a positive attitude in the face of challenges, thereby enhancing better academic performance (Diao et al. 2020; Dong et al. 2021). Thinking transformation is an essential cognitive skill for students to face the real world flexibly, and the information age emphasizes the development of students’ creative thinking more.

In summary, a substantial body of research has demonstrated a robust connection between cognitive ability and academic performance. Although the evidence supporting this relationship is conclusive, there still exists a lack of consensus regarding the complex interactions among the multiple dimensions of cognitive ability, as well as their ensuing effects on academic performance spanning a variety of subjects. Drawing upon the classifications put forward by Wo (2010) and Liang et al. (2020), the current study conceptualizes cognitive ability into five distinct classifications: memory ability (MA), information processing ability (IPA), representation ability (RA), logical reasoning ability (LRA), and thinking conversion ability (TCA). It investigates the influence that these aspects of cognitive ability have on overall academic performance, which is measured by composite scores of Chinese, mathematics, and English. Thereupon, the following hypothesis is posited:

**Hypothesis** **1.** *Cognitive ability can positively predict students’ academic performance.*

### 2.2. The Mediating Role of Self-Control

Metacognitive strategy is defined as the monitoring and regulation of cognitive processes, encompassing such higher-order activities as planning, monitoring, and evaluation. As a core component of metacognitive frameworks, self-control denotes the ability to inhibit immediate impulses and desires in service of long-term goals. From the “independent capability” perspective, self-control may be conceptualized as a stable cognitive function module—closely linked to executive function and grounded in neurobiological mechanisms. Empirically, individuals with high self-control demonstrate sustained resistance to distractions and the independent employment of deep cognitive strategies, in contrast to those with lower self-control, who tend to rely on superficial processing approaches.

From the “learning skill” paradigm, self-control is characterized as trainable strategic behavior, emphasizing the enhancement of self-regulatory efficacy through structured interventions. For example, implementing time management techniques such as the Pomodoro method—whereby learning tasks are divided into manageable intervals—facilitates the gradual acquisition of gratification delay skills. In this scholarly discourse, we opt to conceptualize self-control primarily as an independent capability, distinct from situational regulatory behaviors, to emphasize its role as a foundational cognitive construct with measurable impacts on academic processes.

Self-control involves continuous monitoring and regulation of one’s own behaviors to align with personal values and social expectations (Shang 2019). Self-control is a prerequisite for individuals to continuously pursue progress in academic and life domains. Substantial research has shown that good self-control helps individuals form adaptive habits and promotes academic success and life goal attainment (Chen et al. 2023). Duckworth et al. proposed in their 2019 study that self-control refers to the ability of an individual to stay aligned with long-term goals when facing short-term temptations. This ability plays an important role in academic achievements because students often need to make choices between long-term academic goals and short-term distractions (Duckworth et al. 2019). Whether it is the achievement of long-term or short-term goals, students need to maintain self-discipline and persevere. Especially in the context of cultural differences, most Chinese students spend their time in school life. The school’s rules and regulations structure students and urge them to complete learning tasks on time. Chinese students have relatively high self-discipline and can adhere to their goals and keep working hard to achieve better academic performance. Self-control is recognized as an important factor in the development of children and adolescents, and both the constructs of persistence and self-discipline are key components of self-control (Li et al. 2019). They play a significant predictive role in predicting students’ academic performance and warrant further investigation.

By reviewing a large body of the literature, it is found that few scholars have focused on the trait of persistence; while persistence research exists (Credé et al. 2017), its operationalization in adolescent academic contexts remains limited, particularly in non-Western samples. Studies have shown that the continuous efforts of children in the first grade can affect their future achievements and that persistence is associated with preschool math skills for fourth-grade math and reading scores (Schmerse and Zitzmann 2021). In a recent study with medical students as the participants, the relationship between motivation, academic performance, and persistence was investigated, and it was found that there is a significant positive correlation between persistence and performance achievements (Faisal 2024). Huttunen et al. (2024) found that among adolescents with lower socioeconomic status (SES), students with low academic persistence also had lower school engagement. Although some existing studies have conducted relevant research on the impact of academic persistence on academic achievements, most of them investigate within the context of learning self-efficacy and have not been independently studied in depth. Therefore, in this study, it will be discussed as a key variable separately.

Compared with persistence, self-discipline is defined as an individual’s ability to control the individual’s own behaviors, emotions, and desires. Self-disciplined people can avoid interference from external distractions and short-term temptations. Self-discipline is also considered an important determinant of academic performance. For example, Shi and Qu (2021) observed that students with stronger self-discipline usually perform better academically than their peers, and this finding was confirmed by Hagger and Hamilton (2019). Students with strong self-discipline are less likely to give up or be discouraged when facing difficulties and setbacks academically. Scholars studied the academic performance of K-12 students in North America and found that students’ self-discipline was significantly correlated with their Iowa Tests of Basic Skills (ITBS) scores and GPA, and students’ diligence had the highest correlation with academic performance (Mbaluka et al. 2021). Self-control is crucial for achieving long-term goals. Almost all students will experience conflicts between academic goals and non-academic goals that offer more immediate satisfaction. Self-discipline has a strong correlation with academic achievements, course grades, and performance on standardized achievement tests (Duckworth et al. 2019). There are also studies that tested academic self-discipline as a mediating variable in the relationship between academic self-efficacy and academic performance, drawing on social cognitive theory and self-regulated learning. The studies showed that academic self-discipline is consistently associated with academic performance, including retaining or maintaining one’s GPA (Jung et al. 2017).

Self-control has a significant impact on students’ learning strategies across academic domains. In recent years, studies have found that self-control ability not only directly affects academic achievements but so do academic achievements by influencing learning strategies (Zhu et al. 2018). Specifically, students with high self-control are more likely to adopt effective learning strategies, such as metacognitive strategies and effective strategies, thereby improving their academic self-efficacy and performance (Zhu and Zhang 2021). In addition, the use of self-regulatory strategies, such as situation selection and modification, attention deployment, cognitive change, and response inhibition, can help students manage their learning processes more effectively, improve learning efficiency, and enhance academic performance (Chen et al. 2023). In the online learning environment, self-control is particularly important. Students with higher self-motivation know better how to manage time and plan learning goals, and they will also urge themselves to work hard to achieve these goals, so they often have better academic performance.

Regarding the mechanism of the mediating mechanisms self-control ability, cognitive ability, and academic performance, existing studies suggest that cognitive ability may affect an individual’s academic performance by influencing students’ self-control ability. The scholar Yu (2014) found that individuals with high working memory ability perform better in self-control tasks, not because they have a higher baseline level of self-control resources, but because they tend to invest more energy in the self-control process. Foreign studies have more often explored the mechanism using the framework of self-control depletion. For example, some studies have found that when an individual’s self-depletion—a state of reduced self-control—becomes impaired, it will then affect working memory ability (Singh and Göritz 2018). Lindner and Retelsdorf’s (2020) experiment found that students with a higher degree of self-control depletion had lower task engagement in subsequent tests, were more easily distracted, and achieved lower English scores. These facts suggest that self-control ability is a protective factor for most outcome variables. There are also studies that found that even after controlling for students’ past high school grades and working memory capacity, academic procrastination has a negative impact on subsequent academic performance, thereby shedding light on the role of self-control ability in the relationship between cognitive ability and academic performance (Gareau et al. 2019).

In the existing literature, scholars across the board have commonly acknowledged that there exists a significant correlation between cognitive ability and academic performance. Meanwhile, since self-control ability has been identified as a crucial determinant of academic performance, the relationship between this construct and cognitive ability has inevitably drawn extensive scholarly attention. Against this backdrop, the present study proceeds to put forward the ensuing hypothesis:

**Hypothesis** **2.** *Students’ self-control ability plays a mediating role between cognitive ability and academic performance.*

### 2.3. The Moderating Effect of the Plan

A learning plan focuses on students’ metacognitive framework for exploring and determining learning and career goals, as well as future plans (Hatch 2014). A learning plan is a structured and sequential blueprint aimed at helping individuals acquire new knowledge, skills, or understanding by setting goals, selecting learning methods and resources, and formulating schedules. With the continuous progress of China’s high school curriculum reform, students’ learning methods have to be improved. The formulation of learning plans and the execution ability to operationalize the plans have become very important determinants for high school students to improve their learning ability at present and are demonstrating a robust correlation with students’ academic performance.

“Plan” embodies multifaceted connotations with different definitions and applications in different fields and situations. In educational research, most scholars operationalize the concept of “individual learning plan” or “personalized learning plan” (Individualized learning plan) (ILP), that is, the whole process of sequential stages of knowledge acquisition and smoothly transitioning from one step to another so that students can maintain focus and concentration when learning (Abi Hamid et al. 2018; Suarto 2017). Over 35 states in the United States have mentioned ILP in their educational legislation, with the explicit objective of students being able to achieve elevated academic outcomes through the systematic plan development and implementation of learning plans (Britton and Spencer 2020).

Learning plans have been identified as one of the critical determinants affecting students’ academic performance. Among high school students, those whose grades are consistently in the top 2% often systematically organize their learning lives, and their plans are usually minute-level precision (Ruan 2018). In the context of mathematics education, the scholar Zhao (2021) found in a study that students’ learning planning can significantly enhance their interest and confidence in mathematics learning, as well as their examination scores. In the online teaching context, students with proactive learning planning have lower levels of depression and stress, have more concentration in class, exhibit greater clarity regarding lesson objectives in class, and have higher learning efficiency (Kuang et al. 2023). Through the analysis of the personalized learning behavior data inside and outside the classroom, it is found that there is a positive correlation between students’ personalized learning behaviors and learning achievements, which also indicates that personalized learning plans can optimize academic performance (Yu et al. 2020). In addition, the formulation of learning plans and the formation of behavioral habits have a substantial influence on students’ long-term developmental trajectories and life planning. For example, Britton and Spencer et al. argued in their study that students who complete ILPs are more likely to find careers they are interested in and make informed university admissions decisions according to the institutional fit. Current studies have demonstrated that individuals engaged in professional or learning roles and responsibilities will encounter more work problems and challenges without achieving work–life balance (Gragnano et al. 2020).

However, despite the attention that has been paid to the roles of cognitive ability and self-control, two key gaps remain to be addressed: (1) the independent role of learning plans is unknown: existing research has mostly viewed them as a sub-dimension of self-directed learning strategies (Abi Hamid et al. 2018) and lacks a direct test of plans as a moderating variable; and (2) the mediating mechanism of self-control is unvalidated: whether self-control acts as a mediator between cognitive ability and the achievement role is controversial (Gareau et al. 2019). The present study is the first to simultaneously examine the moderating role of planning in the pathway of “cognitive ability affects self-control, which affects academic achievement” by constructing a moderated mediator model to reveal the mechanism of interaction between the three. Based on these findings, this study proposes the following hypotheses:

**Hypothesis** **3.** *A learning plan plays a moderating role in the impact of self-control ability on academic performance, with higher planning ability strengthening the positive effect of self-control on academic performance.*

Based on goal-setting theory (Locke and Latham 2002), plans may amplify cognitive ability’s effect on self-control and academic outcomes.

**Hypothesis** **4.** *A learning plan plays a moderating role in the impact of cognitive ability on self-control ability.*

**Hypothesis** **5.** *A learning plan plays a moderating role in the impact of cognitive ability on academic performance.*

**Figure 1:**
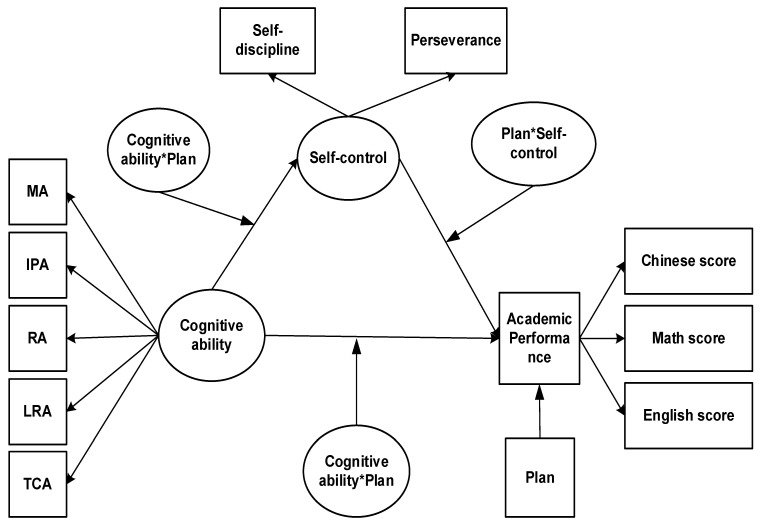
Structural equation relationship moderating: MA, memory ability; IPA, information processing ability; RA, representation ability; LRA, logical reasoning ability; and TCA, thinking conversion ability.

The main research relationships of the structural equation model are shown in Figure 1.

To address these gaps, we tested Hypotheses 3–5 using a latent moderator mediator model, with all constructs operationalized through a multinomial scale validated for reliability and factorial validity.

## 3. Materials and Methods

### 3.1. Participants

The ethical review of this study was approved by the Research Ethics Committee of the School of Humanities and Social Sciences at the University of Science and Technology Beijing. This study selected 1477 students as samples, all aged 15–18 years old, as shown in Table 1.

### 3.2. Procedure

This study seeks to investigate the relationships between students’ cognitive ability, self-control (including persistence and self-discipline), planning ability, and their overall academic performance. To this end, a sample of students was recruited in the campus environment and participated in a battery of two-hour assessments in soundproofed classrooms. Based on the collected data, structural equation modeling (SEM) was employed to analyze the effects of these abilities and the comprehensive academic performance.

During the model analysis stage, the common method bias tests were first administered to evaluate potential biases on each model to ensure the validity and reliability of the data. Subsequently, following standard confirmatory factor analysis (CFA) procedures, the model fit indices were systematically evaluated. After all models met the preliminary fit criteria, cognitive ability and self-control ability were integrated into a mediation analysis framework to explore the mediating roles of these variables in the comprehensive academic performance.

Furthermore, the specific interaction terms examine the potential moderating effects of these interaction terms on the model fit indices. To test the mediating role of self-discipline ability, we adopted the bootstrap method, a resampling technique that provides more robust estimates. Concurrently, simple slope analysis was utilized to explore the moderating role of planning ability, aiming to elucidate how the influence of planning ability on other variables varies across different levels of the moderating variables.

### 3.3. Measures

#### 3.3.1. Personality Traits

In the adopted test system, persistence, self-discipline, and planning were encapsulated under the personality trait dimensions tested with a reliability of 0.762. Confirmatory factor analysis (CFA) adopted a three-factor model of self-control (11 items), cognitive ability (5 items), and learning plan (8 items). Its goodness of fit was significantly better than that of the single-factor model (ΔCFI = 0.182) and the two-factor model (ΔRMSEA = 0.031), and it met the psychometric standards (Chen and Zhao 2016). This result is consistent with the multi-dimensional cognitive structure of the CHC theory and supports construct validity.

##### Self-Control

In the current study, the measurement of self-discipline ability was conducted using the Middle School Students’ Self-discipline Scale developed by Zhang in 2005. This specific scale comprises six items that directly assess dimensions of self-discipline. The self-discipline scale α = 0.79; the persistence scale α = 0.83. In terms of item scoring, a five-point Likert scale was employed. The scoring spectrum spanned from 1 point, denoting “substantially different”, to 5 points, indicating “highly identical”, with the objective of accurately capturing the respondents’ levels of agreement regarding each self-discipline concern. After calculating, the average of the 6 items for each participant was derived and then converted into a *t*-score with a mean of 50 and a standard deviation of 10.

The measurement of persistence ability stemming from the national patent evaluation system was adopted. A sophisticated cognitive assessment system, grounded in stimulus-based methodologies as conceptualized by Wo (2010), was utilized to assess cognitive faculties. This scale functions based on a 5-point Likert scale, wherein the response alternatives span from “strongly disagree” (designated as 1 point) to “strongly agree” (assigned 5 points), and it comprises a total of five items. Once the evaluation process was concluded, the scores procured from each individual question were combined to formulate a comprehensive persistence indicator. Subsequently, this indicator was converted into a *t*-score, which effectively served as the representative metric to denote the persistence ability.

##### Learning Plan

The measurement questionnaire for learning plan ability was carefully constructed by Wo (2010). It was scored using a Likert scale, where 5 represents “very identical”, 4 represents “relatively identical”, 3 represents “uncertain”, 2 represents “relatively different”, and 1 represents “very different”. The scale covers 8 items and comprehensively evaluates students’ planning abilities. During the evaluation process, the scores of students’ responses to each item were accumulated and then converted into *t*-scores, which were used as the students’ planning ability scores. The Cronbach’s alpha coefficient of this scale is 0.88, which indicates that the scale has relatively high internal consistency when measuring planning abilities and ensures the reliability of the evaluation results.

#### 3.3.2. Cognitive Ability

In this paper, the cognitive ability test system of the stimulus information cognitive ability value developed by Professor Wo Jianzhong is adopted, and the system has obtained the Chinese invention patent for this cognitive ability test system (Patent name: Stimulus information cognitive Ability Value test system and its method, patent number: CN101779960B; Patent name: Cognitive index analysis method of potential value test). All 5 dimensions showed good reliability (α = 0.80–0.90). Validated factor analysis (CFA) supported the 5-factor structure (standardized loadings: 0.75–0.92). Cognitive ability was modeled as a latent variable with 5 indicators (MA, IPA, RA, LRA, TCA). Standardized *t*-scores were summed to form composite scores for SEM.

In this study, the stimulus-informed cognitive ability test system developed by Wo (2010) was utilized to evaluate cognitive abilities. This system was integrated with electroencephalogram (EEG) ultra-low-frequency wave analysis technology and ASL504/501 eye-tracking technology to explore the relationship between individual mental processing and brain activity via neural electrophysiological signals and visual attention characteristics.

In terms of experimental design, this study integrated laboratory-based testing with field experiments. Specifically, subtractive and additive response time tasks were utilized to evaluate cognitive processing efficiency, with microcomputer-assisted task administration enabling more precise temporal measurements. These methodological approaches collectively strengthened the discriminative validity and reliability of the test outcomes by minimizing measurement error and enhancing procedural standardization.

Statistical methodologies were employed to systematically assess the cognitive precision of the students under examination. Subsequently, their cognitive ability values were transformed into standardized scores, specifically, *t*-scores, with the aim of ascertaining the cognitive ability scores for each tested individual. This composite score comprised five dimensions, namely, working memory ability (MA), representation ability (RA), information processing ability (IPA), logical reasoning ability (LRA), and thinking conversion ability (TCA). For example, in a representation ability (RA) task, students reconstructed 3D shapes from 2D projections (sample item); in a logical reasoning (LRA) task, they solved syllogisms (e.g., ‘All A are B; some B are C; therefore…’); in a working memory (MA) task, they completed N-back tasks (e.g., press when current digit matches 2-back); in an information processing (IPA) task they performed Digit-Symbol Coding (e.g., match symbols to numbers in 90s); and in a thinking conversion (TCA) task, they engaged in rule-switching tasks (e.g., “Press red for odds → Now press blue for evens”). These dimensions collectively contributed to a comprehensive quantification of the students’ cognitive capabilities, providing a more nuanced understanding of their cognitive profiles. The above test method is protected by an invention patent and has shown significant discriminant validity in the test of a large-sample testing (N > 2,000,000). The results present a normal distribution with a mean of 100 and a standard deviation of 50. In addition, the internal consistency reliability (Cronbach’s alpha) of the scale ranged from 0.80 to 0.90. This result attests to the scale’s high reliability, ensures the stability and consistency of the evaluation process, and provides a solid psychometric basis for the accurate evaluation of students’ cognitive abilities.

This test collected 1477 test data with a reliability of 0.758 and tested for structural validity using Validated Factor Analysis. The results demonstrated that the model fitted well: χ^2^/df = 1.79, CFI = 0.994, TLI = 0.988, RMESA = 0.023, SRMR = 0.015. Its combined validity (CR) = 0.781 and its average variance extracted (AVE) = 0.42, which all meet the requirements.

#### 3.3.3. Academic Performance

In the present study, to mitigate the impact of the influence exerted by the heterogeneity in students’ exam scores, the average of the four examination scores obtained by students during the semester in which their cognitive abilities were assessed was utilized as the academic performance metric for each subject. The raw scores were subsequently standardized, using a linear transformation, to a 0–100 scale. For the purposes of this research, the comprehensive score was established as the primary outcome measure. Specifically, the comprehensive academic performance was calculated as the sum of the scores in Chinese, mathematics, and English, thereby providing a holistic assessment of students’ academic performance in these core subjects.

### 3.4. Data Analysis

Initially, this study investigated the bivariate associations between self-control, cognitive ability, learning plan ability, and academic achievement via Pearson’s correlation analysis. Subsequently, exploratory factor analysis (EFA) was conducted using univariate tests, with factor rotation performed via the varimax method. The extracted factors each exhibited eigenvalues for the three factors all exceeding 1, providing statistical evidence to support the significance of the factor structure. Subsequently, referring to the specification procedures of the moderated mediation effect model proposed by Wen and Ye (2014), the structural equation model was used to conduct in-depth analyses of the mediating effects of persistence and self-discipline, as well as the moderating effect of planning ability. Finally, through simple slope analysis, the specific patterns of the moderating effect were further examined. The data analysis work was conducted using SPSS 25.0 and Mplus 8.3 statistical software to ensure the accuracy and reliability of the analysis results. Through this series of statistical methods, this study aims to reveal the complex dynamic relationships between cognitive ability, persistence, self-discipline, learning plan, and academic achievement.

## 4. Results

### 4.1. Common Method Deviation Test

Before the measurement and statistics, we re-analyzed the data for reliability and validity. We used the McDonald omega statistic for calculation and concluded that the omega coefficient was 0.765 and the validity was 0.803.

To statistically control for potential common method variance (CMV), this study adopted the method of Harman’s single-factor test. When analyzing all constructs items, the unrotated principal component analysis (PCA) method proposed by Podsakoff et al. (2003) was employed in this study.In addition, exploratory factor analysis was performed on the four key variables, namely, self-control, cognitive ability, plan, and academic performance.

The analysis results showed that after factor rotation, the eigenvalues of the three extracted factors were all greater than 1, and the variance explained by the first factor accounts for 29.549%, indicating that the factor structure is statistically significant.

### 4.2. Descriptive and Bivariate Analyses

In this study, a structural equation model was constructed to verify the potential impacts of cognitive ability, self-control (persistence and self-discipline), and plan ability on academic performance. Table 2 presents the results of the model test, in which significant positive correlations are shown between self-control, cognitive ability, plan, and academic performance. The results indicate that these three psychological variables possess important predictive validity in predicting academic achievement.

### 4.3. Measurement Model Verification

In the present study, the structural equation model was strategically employed to systematically examine the pathways in which self-control, cognitive ability, and planning ability influence academic performance. As illustrated in Table 2, the resultant findings revealed significant associations between these variables and academic achievement. Guided by the moderated mediation model specification framework proposed by Wen and Ye in 2014, this research initially analyzed the direct effect of self-control on academic performance. Subsequently, it probed deeply into the mediating role that cognitive ability played within this relational context, suggesting that cognitive ability could potentially act as a crucial conduit bridging self-control and academic performance. Furthermore, the moderating influence of planning ability also fell under the purview of this investigation. In other words, planning ability might either moderate the mediating effect interlinking self-control and academic performance or directly oversee and modulate the relationship that exists between self-control and academic performance.

Firstly, with self-control as the independent variable, comprehensive academic performance as the dependent variable, and self-control ability as the mediating variable, an equation model was established, and it was tested whether the direct effect was moderated by the variable of plan The results demonstrated that the model fitted well: χ2/df = 4.85, CFI = 0.962, TLI = 0.941, RMESA = 0.051, SRMR = 0.030.

In this research, cognitive ability was found to be a positive predictor of comprehensive academic performance, with β = 0.418 and *p* < 0.001. The 95% CI of [0.346, 0.483] further strengthened this relationship. However, regarding the interaction term (cognitive ability × plan) between cognitive ability and plan for comprehensive academic performance, it was not a significant predictor. The regression coefficient b was −0.038 and *p* = 0.107 > 0.05, and the 95% CI of [−0.086, 0.008] contained 0, suggesting no significance. Overall, the direct link between cognitive ability and academic performance was unaffected by plan. Thus, Hypothesis 1 was supported, while Hypothesis 5 was refuted, based on the study data.

**Figure 2:**
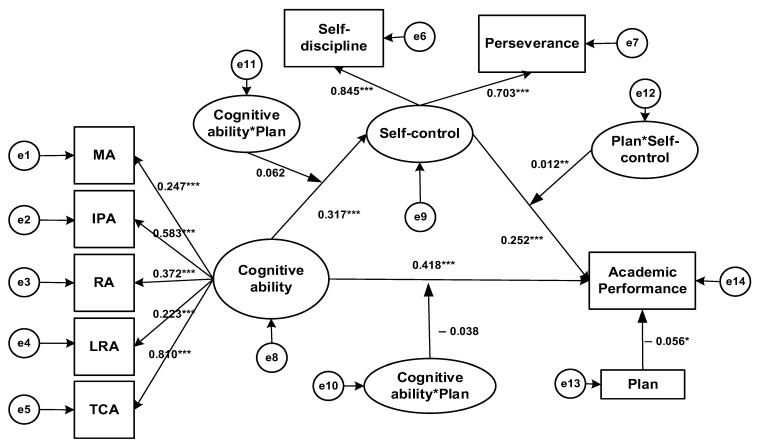
Structural equation modeling results diagram (English Academic Achievement). MA, memory ability; IPA, information processing ability; RA, representation ability; LRA, logical reasoning ability; TCA, thinking conversion ability. * *p* < 0.05, ** *p* < 0.01, *** *p* < 0.001.

Secondly, the impact of cognitive ability on comprehensive academic performance was measured through self-control ability to observe whether the mediating role was significant and whether the moderating role of plan was significant. The results are shown in Figure 2. 

In the current research, self-control ability significantly predicted comprehensive academic performance, with β = 0.252, *p* < 0.001, and 95% CI = [0.172, 0.320]. Cognitive ability could predict self-control: β = 0.317, *p* < 0.001, 95% CI = [0.246, 0.386]. The interaction term (cognitive ability * plan) of cognitive and planning abilities could not predict self-control: β = 0.062, *p* = 0.131, 95% CI = [−0.018, 0.143]. The interaction term (UW) of self-control and planning abilities could predict Y: β = 0.012, *p* = 0.005 < 0.01, 95% CI = [0.0035, 0.021]. The mediating effect of self-control between cognitive ability and Y was significant: mediating effect = 0.402, SE = 0.090, *p* < 0.001, 95% CI = [0.249, 0.603], with the first half unmoderated and the second half moderated by plan. Hypotheses 2 and 3 held, while Hypothesis 4 did not.

**Figure 3:**
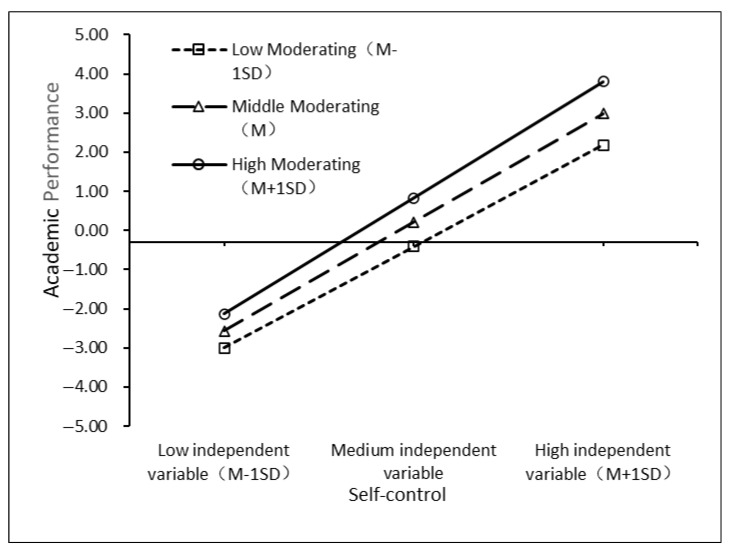
Simple slope test.

To explore plan’s moderating role, students were grouped into “high/low planning” by ± 1 SD. The simple slope test (Figure 3) shows self-discipline predicts the high planning group’s acad. perf. significantly (b = 0.509, *p* < 0.001) and also predicts the low planning group’s (b = 0.497, *p* < 0.001), but weaker. When a mediating process is moderated, it is key to test if the mediating effect changes with cognitive ability. As Edwards and Lambert (2007) suggested, we use cognitive ability values (UH = 1, UL = -1) to check if the mediating effect is differ. between groups, helping to understand complex relations between self-discipline, planning, and acad. perf. The mediating effect of self-discipline in the high planning group is 0.406, with 95% CI = [0.254, 0.612]; the mediating effect of self-discipline in the low planning group is 0.397, with 95% CI = [0.245, 0.599]; and the comparison of the mediating effects is significant (*p* < 0.001), and 95% CI = [0.003, 0.018]. This indicates that under the condition of high planning, the mediating effect is stronger, and the moderated mediation model has been verified.

By using the structural equation model, it is concluded that Hypothesis 1 (cognitive ability can positively predict students’ academic performance) is supported. Hypothesis 5 (learning plan plays a moderating role in the impact of cognitive ability on academic performance) is refuted; Hypotheses 2 (students’ self-control ability plays a mediating role between cognitive ability and academic ability performance) and 3 (learning plan plays a moderating role in the impact of self-control ability on academic performance) held, while Hypothesis 4 (learning plan plays a moderating role in the impact of cognitive ability on self-control ability) did not.

## 5. Discussion

### 5.1. The Relationship Between Cognitive Ability and Academic Performance

Consistent with decades of research (Xu et al. 2023; Zheng 2024; Zhu and Zhang 2021), cognitive ability predicted academic performance (β = 0.418 ***), and self-control mediated this path (effect = 0.402 ***). This study verified for the first time the moderating role of study plans between self-control and academic achievement (H3 established), filling the research gap of ‘unknown mechanism of the role of plans’ pointed out in the introduction. Meanwhile, the mediating effect of self-control (H2 holds) further reveals the psychological pathways through which cognitive ability affects achievement, providing empirical support for the theoretical framework of ‘non-cognitive factors’.

Planning’s moderation effect (b = 0.012 **) reveals a boundary condition: self-control’s impact on achievement strengthens when students systematize efforts via structured plans (Figure 3), supporting Zimmerman’s (2002) self-regulated learning theory.

It can thus be considered that the educational environment and teaching strategies in the middle school stage are vital for cultivating students’ cognitive abilities, and the improvement of these abilities directly affects students’ academic performance.

### 5.2. The Mediating Role of Self-Control Ability

The research results indicate that self-control ability serves as a mediating factor between cognitive ability and academic performance; specifically, self-control ability is an important critical mechanism through which cognitive ability affects academic performance. The two key components of self-control are persistence and self-discipline. Persistence reflects an individual’s continuity and effort level in specific tasks. Self-disciplined people can formulate plans and rigorously execute them, avoiding being interfered with by the outside world and short-term temptations. These abilities have substantial predictive power in predicting students’ academic performance. The middle school stage is a crucial period for students to develop their individual cognitive abilities. The escalation of academic complexity and frequent examinations not only promote the improvement of students’ cognitive abilities but also have a profound influence on students’ learning strategies and behavioral patterns, which is further manifested in academic performance.

Self-control not only affects the overall academic performance but may also exert differential effects across academic domains. For example, learning in the STEM fields may particularly place unique demands on self-control to solve complex problems and conduct continuous exploration, while learning in the humanities and social sciences may depend more on the application of self-control in reading and writing tasks (Duckworth et al. 2019). For instance, students with strong attention and memory abilities may be better able to resist distractions and focus on learning tasks. Through persistence and self-discipline, they can continuously improve their academic performance. For example, students with strong working memory ability will consistently engage with vocabulary memorization tasks for English words every day, and within the equivalent time frames, they will maintain focus amid distractions more than other students, and their English scores will also be significantly higher than those of their competitors. In addition, they may be more able to formulate effective learning strategies, such as reasonably arranging learning time and selecting appropriate learning materials and methods, all of which are critical for academic goal attainment. Students with strong cognitive abilities are more likely to adopt effective learning strategies, such as time management, goal setting, and organizational planning. Through self-discipline and persistence, they can continuously incrementally achieve short-term objectives and thus achieve long-term goals.

In summary, by improving self-control ability, students can manage their learning processes more effectively and thus achieve better academic achievements in various subjects. When cognitive ability plays a role through the improvement of self-control ability, students may achieve better results in their studies. This is because they can absorb and process classroom information more effectively, prepare for exams better, and participate in classroom discussions and activities more effectively. Self-control ability is a key factor affecting students’ academic performance, and its influence runs through all aspects of the learning process.

### 5.3. The Moderating Effect of the Plan

Unlike the introduction, which states that ‘planning studies mostly incorporate self-directed learning strategies’, the present study independently examined its moderating role, confirming that planning is a key contextual factor in the relationship between self-control and achievement rather than an accessory strategy. A learning plan is an arrangement for learning activities that learners make in a planned and step-by-step manner, according to their actual situations and learning contents, in order to achieve certain learning goals. This study found that the learning plan does not play a moderating role in the process of the impact of cognitive ability on academic performance or in the process of the impact of cognitive ability on self-control ability, that is, Hypotheses 4 and 5 do not hold. However, we found that the learning plan plays a positive moderating role in the process of the impact of self-control ability on academic performance. This result may stem from the fact that plans are essentially goal execution tools that rely on self-control for their realization (Duckworth et al. 2019). High planners are able to translate self-control into efficient actions (e.g., strict execution of schedules), whereas cognitive ability as a stabilizing trait (e.g., working memory capacity) is less susceptible to plan interventions (H4, H5 do not hold true), which explains why plans did not modulate cognitive ability-related pathways.

Domestic Chinese research, based on large-scale surveys of secondary students in several middle schools in a certain region, found that self-control ability is positively correlated with academic performance, and the use of learning strategies also affects academic performance to a certain extent. Structural equation modeling found that the learning plan, as an important part of metacognitive strategies, has a positive moderating effect on the relationship between self-control ability and academic performance (Zhang 2023). Cross-regional research outside China also shows that students who make learning plans perform better in higher levels of self-regulated learning. Their learning is more systematic and efficient, and their academic performance is also significantly higher than that of students who do not make learning plans (Xiao et al. 2019). The learning plan helps students conduct self-regulated learning better, thereby improving academic performance. This conclusion indirectly supports the view that the learning plan amplifies the predictive effect in the process of the impact of self-control ability on academic performance. The learning plan plays a positive moderating role in the process of the impact of self-control ability on academic performance by helping students clarify learning goals, reasonably arrange learning time, and improve learning efficiency. Students who make learning plans perform better in self-regulated learning. Their learning is more orderly and efficient, and their academic performance is also significantly higher than that of students who do not make learning plans.

The construction of a learning plan not only promotes the improvement of students’ self-monitoring competencies but also enhances their persistence and adaptive problem-solving skills when facing learning difficulties. By setting clear learning goals and timetables, students can manage their learning processes more effectively and adjust metacognitive strategies in a timely manner to cope with challenges. This flexibility and self-regulation ability are crucial for sustaining long-term academic progress. Moreover, students who can effectively implement learning plans often have stronger intrinsic motivation and higher self-efficacy. They are more inclined to regard learning tasks as opportunities for self-actualization rather than externally imposed burdens. This proactive learning orientation further promotes their academic achievements and mental health. Importantly, the effectiveness of a learning plan is not fixed. It is affected by multiple factors, such as individual differences (such as learning styles, cognitive preferences) and external environments (such as familial support and institutional resource availability). Therefore, how to formulate personalized learning plans according to personal characteristics and how to cultivate and support this ability to formulate plans in school and family environments have become important directions for future research and practice. Accordingly, educators can optimize instruction through two strategies that reinforce the performance-promoting effects of self-control through plan-setting training (e.g., the SMART goals approach) and prioritize the enhancement of self-control (e.g., delayed gratification training) over direct intervention in the cognitive ability of students with weak cognitive abilities.

## 6. Limitations and Future Directions

The present study has some limitations in exploring the relationships between self-control, cognitive ability, study plan, and academic performance that need to be noted in future research. First, despite the wide sample, it may have lacked the representativeness of specific groups, such as students from different cultural backgrounds, which limits the generalizability of the findings. Second, this study only used study plan as the only moderating variable, which may not be sufficient to fully explain the complex relationship between self-control and academic performance. Future studies should also consider other potential moderating variables such as academic motivation and emotional state. In addition, the limited number of test questions on self-control and study plan may have restricted the full capture of these potential constructs. Although the reliability coefficient (α > 0.80) is acceptable, future research should incorporate more indicators (such as 8–12 items) into each factor to enhance the representation and generalization of the structure. In subsequent studies, we will break down cognitive ability into different sub-dimensions and conduct in-depth research on the interaction mechanism between different sub-dimensional abilities and personality traits, as well as psychological characteristics. Finally, although cognitive ability measures show robust psychometric properties, they currently rely on a single patent-based system, and future studies need to validate them using a diverse combination of cognitive tests (e.g., Wechsler Adult Intelligence Scale WAIS, Cattell tests). This test is protected by China’s Intellectual Property Patent Law; if readers wish to use this test, they must obtain the consent of Professor Wo Jianzhong’s team before using it. In summary, future research should further reveal the complex dynamics of these psychological variables in education through broader samples, more in-depth variable exploration, and more advanced research methods.

## 7. Conclusions

This research analyzed self-control’s mediating role between cognitive ability and academic performance and verified learning plan’s moderating role via the structural equation model. The findings reveal the following: cognitive ability positively impacts academic performance; self-control mediates; and plan’s moderating role is significant in the latter half of mediation. As the plan level rises, self-control’s impact on academic performance changes, becoming stronger at higher plan levels. Thus, the moderated mediation model is established for understanding the variable interplay in an academic context. At the practical level, this study elucidates the mediating role of self-control between cognitive ability and academic achievement, as well as the moderating role of learning planning. These insights offer significant implications for educational practice, psychological counseling, family education, and the formulation of educational policies.

## Figures and Tables

**Table 1:** Distribution of participating students.

Grade	Number of Students			
	Boys	Proportion	Girls	Proportion
First grade	240	49.90%	241	50.10%
Second grade	256	50.39%	252	49.61%
Third grade	268	54.92%	220	45.08%
Total	764	51.73%	713	48.27%

**Table 2:** Means, standard deviations, and intercorrelations for variables.

	M	SD	1	2	3	4	5	6	7	8	9	10	11
**1. Ma**	107.12	17.697	1										
**2. Ipa**	104.44	13.759	0.144 **	1									
**3. Ra**	106.23	7.044	0.025	0.243 **	1								
**4. Ira**	103.78	8.911	0.042	0.135 **	0.097 **	1							
**5. Tca**	97.42	17.391	0.180 **	0.471 **	0.323 **	0.160 **	1						
**6. Persist**	103.32	15.621	0.061 *	0.126 **	0.067 **	0.060 *	0.159 **	1					
**7. Self-control**	106.18	15.678	0.069 **	0.186 **	0.105 **	0.103 **	0.202 **	0.593 **	1				
**8. Plan**	100.23	17.081	−0.011	0.031	0.013	0.036	0.051	0.556 **	0.412 **	1			
**9. T (Chinese)**	50.71	9.938	0.159 **	0.196 **	0.054 *	0.118 **	0.338 **	0.152 **	0.219 **	0.054 *	1		
**10. T (Mathematics)**	50.66	10.014	0.209 **	0.209 **	0.122 **	0.167 **	0.289 **	0.220 **	0.254 **	0.075 **	0.256 **	1	
**11. T (English)**	50.66	9.945	0.186 **	0.184 **	0.028	0.083 **	0.272 **	0.144 **	0.185 **	0.068 **	0.395 **	0.240 **	1
**12. Total**	151.9107	21.79016	0.253 **	0.269 **	0.093 **	0.168 **	0.411 **	0.236 **	0.301 **	0.090 **	0.754 **	0.686 **	0.747 **

Note: *N* = 1477. MA, memory ability; IPA, information processing ability; RA, representation ability; LRA, logical reasoning ability; TCA, thinking conversion ability. * *p* < 0.01, ** *p* < 0.001.

## Data Availability

The original contributions presented in this study are included in the article and Supplementary Materials. Further inquiries can be directed to the corresponding author.

## Supplementary Materials

The following supporting information can be downloaded at: https://www.mdpi.com/article/10.3390/jintelligence13080092/s1, Table S1: DATA1.
